# Glibenclamide Prevents Diabetes in NOD Mice

**DOI:** 10.1371/journal.pone.0168839

**Published:** 2016-12-22

**Authors:** Smaragda Lamprianou, Conny Gysemans, Joanna Bou Saab, Helena Pontes, Chantal Mathieu, Paolo Meda

**Affiliations:** 1 Departement of Cell Physiology and Metabolism, University of Geneva, Geneva, Switzerland; 2 Laboratory of Clinical and Experimental Endocrinology, Katholieke Universiteit Leuven (KULEUVEN), Leuven, Belgium; University of British Columbia, CANADA

## Abstract

Previous work has revealed that Cx36, the sole connexin expressed in the insulin-producing beta cells, enhances the secretion of insulin, and promotes the resistance of beta cells against pro-inflammatory cytokines. In parallel, the anti-diabetic sulphonylurea glibenclamide was shown to promote the assembly and function of Cx36 channels. Here, we assessed whether glibenclamide could protect the insulin-producing cells against conditions mimicking those expected at the onset of type 1 diabetes. We found that the drug 1) protected *in vitro* the mouse MIN6 cells from the apoptosis and loss of Cx36, which are induced by Th1 cytokines; 2) prevented the development of hyperglycemia as well as the loss of beta cells and Cx36, which rapidly develop with aging in untreated NOD mice; 3) modified the proportion of effector CD4^+^ and CD8^+^ T cells in pancreatic draining lymph nodes. The data imply that an early glibenclamide treatment may help protecting beta cells against the autoimmune attack, which triggers the development of type 1 diabetes.

## Introduction

Glibenclamide (glyburide) is an antidiabetic sulfonylurea, which triggers insulin secretion mostly by binding to the regulatory SUR1 subunits of the ATP-sensitive potassium channels of pancreatic beta cells [1]. Glibenclamide also promotes islet expression and function of connexin 36 (Cx36) [2–5], a gap junction protein which significantly contributes to control the secretion and survival of pancreatic beta cells, including under conditions which are thought to prevail in the islet environment at the onset of autoimmune type 1 diabetes [6].

In view of these findings, we first explored *in vitro* the effect of glibenclamide on the insulin-producing cells of the mouse MIN6 line, during the induction of apoptosis by diabetogenic Thelper (Th)-1 cytokines. Previous reports have suggested that sulphonylureas may mitigate the hyperglycaemia which develops with age in the non-obese diabetic mice (NOD), a widely used model of type 1 diabetes [7,8]. However, these studies have also provided conflicting evidence about such a protective role [9–11]. Thus, in a second part of this study, we longitudinally monitored NOD mice during a chronic exposure to glibenclamide, starting at an age when the pathological and biological signs of hyperglycemia and diabetes had not yet developed [12]. While the main focus of this study was to explore whether any protective effect of glibenclamide could be related to its effects on Cx36 signalling, we also explored whether these effects could involve changes in the autoimmune responses of the NOD mice.

Here, we report that glibenclamide 1) protected the mouse insulinoma MIN6 cells against the apoptosis and loss of Cx36, which are induced by pro-inflammatory cytokines; 2) protected diabetes-prone NOD mice, in a dose-dependent manner, against the progressive development of hyperglycemia, as well as the loss of insulin-producing beta cells and of Cx36 expression; 3) did not stop insulitis progression, but induced a shift in the phenotype of immune cells remaining in the pancreatic draining lymph nodes to a CD44^hi^CD62L^-^ effector profile. These findings open the exciting possibility that, by enhancing Cx36 signalling and modulating the autoimmune response, glibenclamide could help promoting the *in vivo* survival of beta cells, under diabetogenic conditions.

## Materials and Methods

### *In vitro* experiments

**Fig 1:**
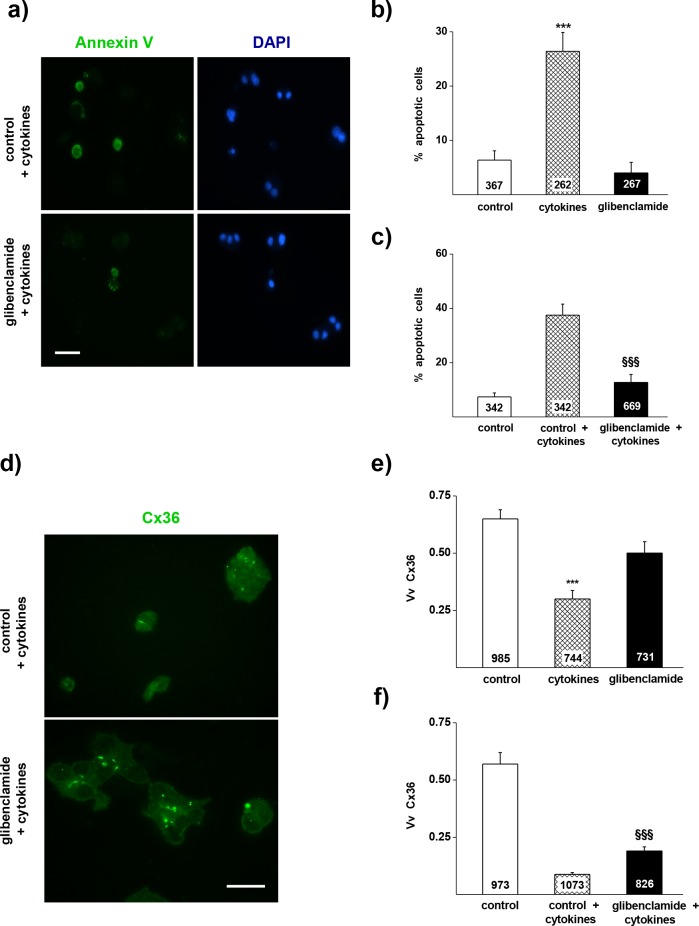
Glibenclamide reduces the apoptosis and the loss of CVx36 in MIN6 cells exposed to Th1 cytokines. **a)** Apoptosis of MIN6 cells was evaluated by the proportion of Annexin V-stained cells (green) out of the total number of cells screened (identified by the blue DAPI staining of nuclei). Bar, 25 μm. **b)** A 18h exposure of MIN6 cells to a mix of IL-1β, IFN γ and TNFα induced a significant increase in the proportion of Annexin V-labelled cells (hatched bar), compared to the level observed in controls cultured in the absence of cytokines (open bar). Such an increase was not observed in cells exposed to 10 μM glibenclamide (solid bar). **c)** Comparable effects were seen when MIN6 cells were exposed to 10 μM glibenclamide 6 h before starting the exposure to the cytokines. **d)** Expression of Cx36 was evaluated by immunostaining, using an antibody that detects the small gap junction plaques made by the concentration of Cx36 within the membrane (yellow-green). Bar, 5 μm. **e)** A 18h exposure of MIN6 cells to IL-1β, IFN γ and TNFα (hatched bar) reduced the volume density of immunolabeled Cx36 under the control levels (open bar). Such a decrease was not observed in cells exposed to 10 μM glibenclamide (solid bar). **f)**. Comparable effects were seen when MIN6 cells were exposed to 10 μM glibenclamide 6 h before starting the exposure to the cytokines. Data are mean + SEM of 3 independent experiments. Numbers within bars indicate the total number of cells scored in each group. *** p < 0.001 vs control group;. ^§§§^ p < 0.001 vs control + cytokines group, as tested by one-way ANOVA.

MIN6 cells (passages 5–10) were obtained from Dr. Jun-Ichi Miyazaki (School of Medicine of Kumamoto University, Kumamoto 862, Japan) and, thereafter, were passed weekly. For this study, the cells were cultured for 3 days as described [2,3]. At this time, the medium was replaced with either fresh DMEM supplemented with 0.1% DMSO (control group), DMEM supplemented with 0.1% DMSO and 10 μM glibenclamide (glibenclamide group), or DMEM supplemented with 0.1% DMSO, 0.25 ng/ml IL-1β, 9.1 ng/ml TNF-α, and 10 ng/ml IFN-γ (cytokine group), and the cultures were grown for 18 h. The cytokine concentrations were chosen from previous studies [6–8,13] and kept to the minimum levels producing a significant apoptosis, in order to minimize pleiotropic effects. In a second set of experiment, cells were first exposed for 6 h to either the control or the glibenclamide groups, and then exposed for 18 additional hours to the conditions of the cytokine group (control + cytokines and glibenclamide + cytokines groups, respectively). At the end of all experiments, a part of each sample was processed for quantitative analysis of Cx36 immunolabelling [2,3,8]. Briefly, cells were permeabilized for 3 min in -20°C acetone, rinsed in phosphate-buffered saline (PBS) containing 2% bovine serum albumin (BSA), incubated for 2h at room temperature with rabbit polyclonal antibodies against Cx36 (51–6200, Invitrogen, Weltham, MA, USA), and again for 1h at room temperature with Alexa 488-labelled donkey polyclonal antibodies against rabbit Igs (A-11034, Invitrogen, Weltham, MA, USA). The specificity of both antibodies has been established [2,3,8]. Here, this specificity was assessed by i) the positive, dotted staining of many control MIN6 cells, ii) the consistently positive staining of primary beta cells in control pancreas sections; iii) the negative staining, in the same sections, of islet non beta cells, exocrine acinar cells, immune, connective and vascular cells of pancreas; iv) the loss of all immunostaining after omission of the primary antibody and incubation of cells and sections with only the secondary antibodies. Cover slipped cultures were photographed with an Axiophot epifluorescence microscope equipped with an AxioCam camera (Carl Zeiss, Oberkochen, Germany). Digitized images were scored using the Image J software (NIH, Bethesda, MD, USA) for the areas of fluorescent Cx36 spots and of the corresponding cells. The volume density (Vv) of Cx36 was given by the ratio of these two sets of areas [2,3]. Other culture dishes were processed for evaluation of apoptosis [3,14], using an Annexin V-EGFP Apoptosis Detection Kit (BioVision, ref. #: K104-100, Milpitas, CA). Briefly, cells were incubated for 5 min in the dark and at room temperature with Annexin V-EGFP in binding buffer, washed, fixed in 2% paraformaldehyde (PFA) and cover slipped with a DAPI-containing solution. Apoptotic cells that had bound Annexin V-EGFP showed a green plasma membrane staining (Fig 1A) and could be scored, on fluorescence images taken at random in the culture dishes. The apoptosis incidence was given by the percentage of Annexin V-EGFP-positive cells over the total number of cells scored, which was given by the number of nuclei stained in blue by DAPI (Fig 1A).

### *In vivo* experiments

Inbred non-obese diabetic (NOD) female mice, initially obtained from Dr. Wu (Peking Union Medical College hospital, Beijing, China), were bred under semi-barrier conditions at the KULEUVEN animal facility since 1989, according to the FELASA guidelines. The diabetes incidence of this colony varies between 50–70% in female mice at 30 weeks of age. Animals were maintained in accordance with the Guide for the Care and Use of Laboratory Animals (NIH, Bethesda, USA), and the experiments were approved by the KULEUVEN Ethical Committee (file P267/2014). Animals were housed in grid cages to facilitate the scoring of glycosuria, and monitored 2–3 times a week. At 3 weeks of age, littermates were randomly attributed to 3 different groups: one group (control) did not received treatment; the second group (dose 1) was s.c. implanted with a pellet releasing 0.1 μg glibenclamide over a period of 12 weeks; the third group (dose 2) was similarly implanted with a pellet releasing 10 mg glibenclamide over the same time period. The two glibenclamide concentrations were selected from previous studies [2–5, 12–15] to cover a large dose-response range. According to the manufacturer’s information (http://www.innovrsrch.com/pellets.asp), each pellet used in group 2 released 9–12.6 mg glibenclamide per day in a 25–35 g b.w. mouse, a doses which favorably compares with the maximal doses of 15 mg glibenclamide/day recommended in human patients [14]. All implanted mice received a second pellet 12 weeks later, to ensure steady drug levels, as per the manufacturer’s indications. The KULEUVEN investigators who run the animal part of the study were blinded to both the nature of the drug investigated, and the doses administered.

The animals were then longitudinally monitored for body weight, glucosuria, glycaemia, and, in week 25^th^, were submitted to a glucose tolerance test, after an i.p. injection of 2.2 g D-glucose / kg b.w. Mice that became diabetic (glycosuria, 2 consecutive measurements of blood glucose >200 mg/dl, loss of more than 20% b.w.) were sacrificed. The experiment was terminated on week 25 (28 weeks of age), at which time organs and blood were sampled from all surviving animals. After anesthesia under 5% isoflurane (Rhodia Organique Fine LTD., Avonmouth, Bristol, UK), mice were killed by cervical dislocation and exsanguinated by heart puncture. The pancreas (on week 25), pancreatic lymph nodes and spleen (on weeks 3 and 6) were removed under aseptic conditions. After rinsing in PBS, part of the pancreas was immediately frozen in liquid nitrogen and stored at -80°C for immunofluorescence. Other pancreatic fragments were fixed in 4% PFA for morphometry.

### Pancreas analysis

Pancreas fragments from at least 3 mice of each group were embedded in paraffin. Serial sections were stained with haematoxylin-eosin (HE) to evaluate insulitis or immunostained with antibodies against insulin to quantitate beta cells [2,3,15]. In the latter case, sections were fixed 15 min in 4% PFA, incubated 30 min in 10 mM NH_4_Cl in PBS, exposed 30 min to a 2% BSA solution in PBS, incubated 2 h at room temperature with an anti-insulin guinea pig antibody (Ventrex 675) diluted 1/200, exposed for 1 h to a goat anti-guinea pig-FITC (1/400, P.A.R.I.S, Biotech, Paris, France), and covered with a DAPI-containing solution. Immunolabeled sections were scanned using a MIRAX MIDI slide scanner (Carl Zeiss, Oberkochen, Germany), operated under UV illumination, and analysed using the MIRAX Viewer software to evaluate the volume density of beta cells and insulitis. Briefly, the outlines of each islet and insulitis mantle were drawn by hand on projections of the digitized images, and the areas of islet profile, insulin-expressing beta cells and insulitis scored. Values of islet numbers and beta cell areas were expressed relative to the area of each pancreatic slice, to estimate numerical (Nv) and volume densities (Vv), respectively. Cryostat sections (7 μm thickness) of pancreas fragments were processed for immunostaining of Cx36, and quantitatively evaluated, as described above for MIN6 cells.

### Flow cytometric T cell analysis

Single cell suspensions were prepared from spleen and pancreatic-draining lymph nodes of untreated controls and dose 2 glibenclamide-treated mice, at 3 and 6 wks of treatment. Cells were stained with fluorochrome-labelled antibodies against CD3e (145‐2C11), CD4 (GK1.5), CD8a (53–6.7), CD25 (PC61), CD44 (IM7), CD62L (MEL-14), and matching isotype controls (all antibodies from eBioscience, San Diego, USA). Foxp3 staining was further performed using an eBioscience staining kit, according to the manufacturer’s instructions. Cells were analysed on a Gallios™ flow cytometer (Beckman Coulter, Analis, Suarlée, Belgium), using a gating strategy that separated cells based on live CD3^+^ lymphocytes. The proportion of Tregs in the CD4^+^ lymphocyte gate was calculated based on the combination of staining for cell surface markers (CD4, CD25) and for the intracellular transcription factor forkhead box protein 3 (Foxp3). CD4^+^ and CD8^+^ T cells were further attributed to the naïve, effector and memory groups, based on the expression of the adhesion molecules CD44 and CD62L: naïve cells: CD44^lo^CD62L^+^; effector cells: CD44^hi^CD62L^–^; memory cells: CD44^hi^CD62L^+^ (S1 Fig). Data were analysed by the FlowJow software TreeStar.

### Statistical analysis

Results were expressed as means + SEM when the data were normally distributed, and as medians when the data showed an asymmetric distribution. Accordingly, the levels of significance between groups were assayed by t-test and analysis of variance (ANOVA) for normally distributed values, and by the Mann-Whitney and median tests for asymmetrically distributed values, as provided by the GraphPad Prism6 software (La Jolla, San Diego, CA). The percentage of mice featuring normal and altered glycaemia was plotted as a function of time, and compared with the Mantel-Cox log-rank test.

## Results

### Glibenclamide decreases the apoptosis and loss of Cx36 of MIN6 cells exposed to cytokines

Apoptosis of MIN6 cells was evaluated by Annexin V-EGFP labelling (Fig 1A). As compared to the proportion of apoptotic cells cultured under control medium, a 18h exposure to a mix of IL-1β, IFN-γ and TNF-α significantly increased the Annexin V staining of MIN6 cells (Fig 1B). Such an increase was not observed in the presence of 10 μM glibenclamide (Fig 1B). In the very same experiments, the expression of Cx36 was evaluated by immunostaining (Fig 1D). Exposure to the cytokines significantly decreased the control volume density of Cx36, a decrease which was prevented in the presence of glibenclamide (Fig 1E). Comparable effects were observed when the cells were exposed to the drug 6h before the exposure to the cytokine mix (Fig 1C and 1F).

In view of the alterations observed in the murine MIN6 cells exposed to glibenclamide, we decided to investigate the effects of the drug in NOD mice, a well accepted model of autoimmune type 1 diabetes [7,8].

### The incidence of glucose intolerance and diabetes differs in control and glibenclamide-trated NOD mice

**Fig 2:**
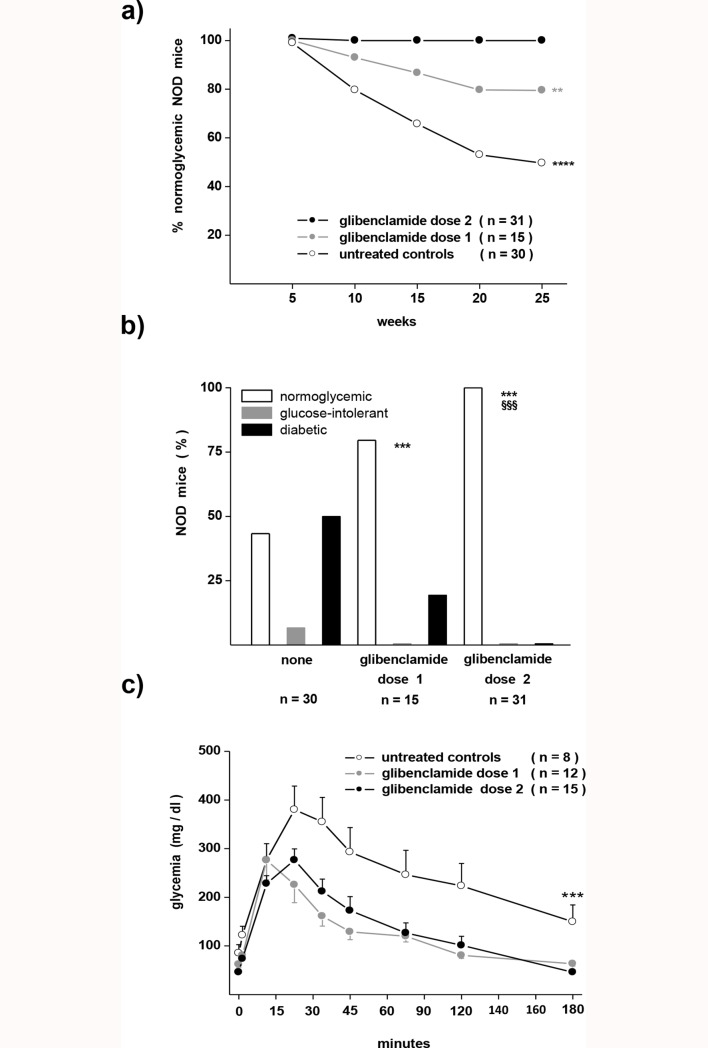
Glibenclamide treatment prevents the development of diabetes in NOD mice. a) All mice had normal blood glucose levels at week 5. Thereafter, the proportion of untreated animals, which remained normoglycaemic, rapidly dropped with time (open symbols). In contrast, all littermates that received the high dose of glibenclamide (glibenclamide dose 2, black symbols) remained normoglycaemic till the end of the experiment. Mice that received the lower dose of glibenclamide (glibenclamide dose 1, grey symbols) featured an intermediate evolution. ** p< 0.01,**** p< 0.0001 vs control group, as tested by the Mantel-Cox log-rank test. n are the numbers of animals tested in 1 (glibenclamide dose 1 group)-2 (control and glibenclamide dose 2 groups) experiments. **b)** Bars show the percentage of NOD mice featuring normoglycaemia (open bars), glucose intolerance (grey bars) and overt diabetes (solid bars) at the end of the 25 wk experiment. n are numbers of mice investigated in 1 (glibenclamide dose 1 group)-2 (control and glibenclamide dose 2 groups) experiments. No error bars are given in panels a) and b) given that these graphs show a single percentage value calculated for the entire mouse population tested. ***p< 0.001 vs control group, ^§§§^p < 0.001 vs glibenclamide dose 1 group, as tested by the Chi square test. **c)** After a glucose challenge untreated mice surviving till the week 25 (open symbols) displayed a sustained hyperglycaemia indicating glucose intolerance, whereas the glibenclamide-treated mice rapidly returned to normoglycaemia. Data are mean + SEM values of glycaemias from an experiment that tested in parallel the 3 groups of NOD mice. *** p< 0.001 compared to the glibenclamide-treated groups, as tested by ANOVA. n are numbers of mice investigated.

The control group of female NOD mice, which received no treatment, developed within 6 weeks of age an altered control of glycaemia, which was not observed in littermate groups of female NOD mice that received 20 mg glibenclamide (dose 2), from weaning onwards (Fig 2A). The littermate group of female NOD mice that received 0.2 μg glibenclamide weeks (dose 1) for the same time period, featured an intermediate phenotype (Fig 2A). At the end of the experimental period (week 25), 50% of the untreated mice became overtly diabetic (Fig 2B), with an additional 7% exhibiting glucose intolerance (Fig 2B and 2C). Conversely, the animals receiving either dose 1 or dose 2 of glibenclamide were protected against these alterations (Fig 2B and 2C). At the end of the experimental period, none of the mice of the glibenclamide dose 2 group had developed glucose intolerance or overt diabetes (Fig 2A–2C). In the glibenclamide dose 1 group, 20% of the mice developed overt diabetes, and none featured glucose intolerance (Fig 2A–2C).

### The glibenclamide treatment decreases the spontaneous loss of islets, and preserves Cx36 expression in beta cells of NOD mice

**Fig 3:**
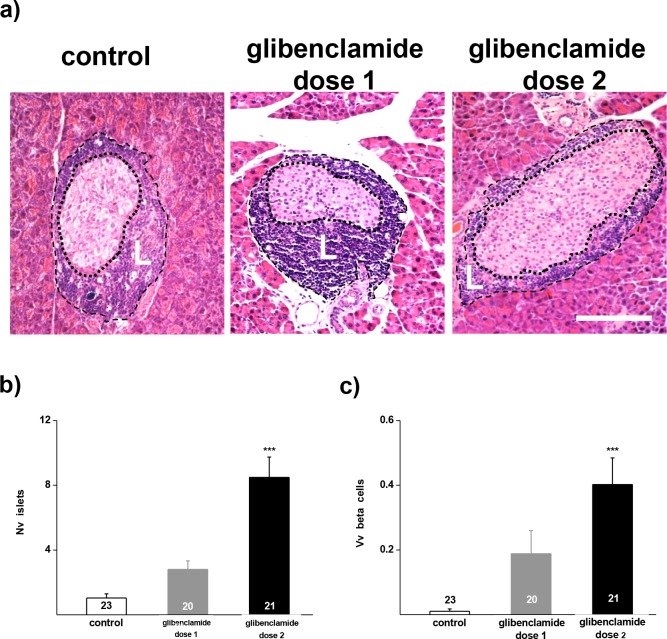
Glibenclamide preserves a sizable mass of beta cells in adult NOD mice. **a)** HE staining of pancreas sections shows representative islets of the 3 groups of NOD mice, which we compared. At the end of the experiment, untreated control mice aged 28 wks showed few islets with many of the residual cells featuring a poorly stained cytoplasm. In contrast, mice treated with either dose 1 or dose 2 glibenclamide featured many more and larger islets, which mostly comprised healthy looking cells. Insulitis was prominent in the islets of all mice groups. The dot and dash lines outline the borders of islets and insulitis mantle (L), respectively. Bar, 100 μm. **b)** At the end of the experiment, the numerical density (Nv) of beta cells was significantly higher in the glibenclamide dose 2-treated NOD mice (solid bars), than in untreated controls (open bars). The mice receiving dose 1 of glibenclamide featured an intermediate phenotype. **c)** The volume density (Vv) of beta cells was similarly modified by the glibenclamide treatments. Data are mean + SEM values of the number of pancreas sections indicated within the bars. * p< 0.05, ** p< 0.01, *** p< 0.001 vs control group, as tested by ANOVA.

At the end of the experiment (25 wk), islets were observed in the pancreas of the three groups of mice which we compared (Fig 3A). However, these residual islets were rare in the control, untreated group of hyperglycaemic mice, and much more frequent in the two groups of glibenclamide-treated animals (Fig 3A). In the group of untreated control mice, the alterations in glycaemia associated with a decrease in the numerical density of islets (Fig 3B) and in the volume density of the insulin-containing beta cells which, at the end of the experiment, represented <0.05% of the pancreas volume (Fig 3C). A much lower decrease in the densities of islets and beta cells was observed in mice of the glibenclamide dose 2 group (Fig 3B and 3C). The mice of the glibenclamide dose 1 group featured an intermediate phenotype, with values of beta cell densities between those evaluated in the untreated controls and in the mice of the glibenclamide dose 2 group (Fig 3B and 3C).

**Fig 4:**
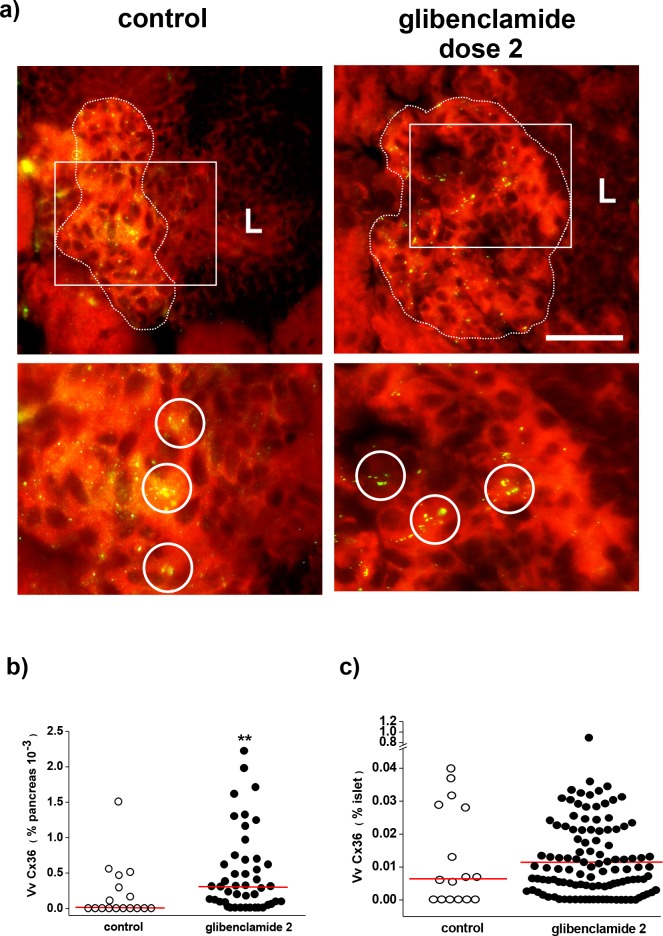
Glibenclamide treatment preserves Cx36 expression in beta cells. **a)** At the end of the 25 wk experiment, immunostaining reveals the spotted distribution of Cx36 (yellow) in the few residual islets of control, untreated mice and in the more numerous islets of the glibenclamide dose 2-treated mice. The enlargements of the field outlined in the pictures show that Cx36 formed larger and more numerous spots (some are circled) in the islets of the latter animals. The dot line outlines the limit between an islet and its surrounding insulitis mantle (L). Bar, 40 μm in the pictures, 20 μm in the enlargements. The red background staining is due to the Evans blue counterstain. **b)** The volume density of Cx36 was significantly lower in the whole pancreas of untreated controls than in that of the glibenclamide-treated mice, due to the much larger number of islets found in the latter animals. **c)** In contrast, no significant difference in the volume density of Cx36 was reached in the residual islets of the 2 groups of mice that we compared, possibly because few surviving islets could be scored in the pancreas of the untreated NOD mice. ** p< 0.01 vs control group, as tested by Mann-Whitney and median tests. Median values are shown by the red lines.

At the end of the experiment, Cx36 was detected by immunostaining in the residual islets of both control and glibenclamide dose 2 groups (Fig 4A). However, the volume density of immunolabelled Cx36 was higher (p< 0.01) in the whole pancreas of mice treated with dose 2 of glibenclamide than in that of the untreated animals (Fig 4B), consistent with the preservation of a much larger number of beta cell-containing islets in the former group of animals. At the level of individual islets, the volume density of Cx36 appeared usually lower in the untreated than in the glibenclamide dose 2 group (Fig 4A). However, at the end of the experimental period, this difference failed to reach statistical significance (Fig 4C), due to the low number of residual islets in the pancreas of the untreated NOD mice.

### The glibenclamide treatment does not prevent insulitis, and enriches subsets of effector T cells draining the pancreas

**Fig 5:**
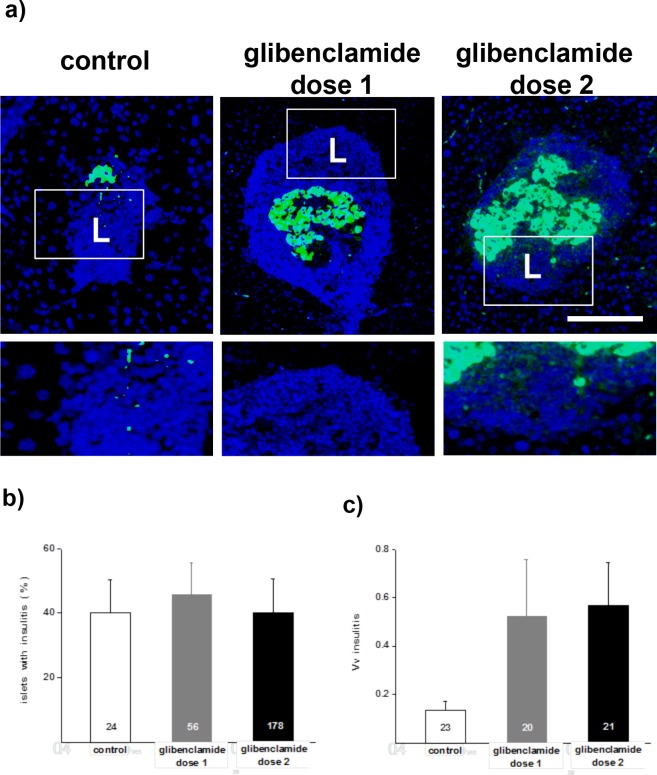
Islets of untreated control and glibenclamide-treated NOD mice display insulitis. **a)** Few beta cells are immunostained for insulin (green) in the islets of untreated controls, used for morphometry, whereas many more beta cells are seen in the islets of the animals treated with glibenclamide. The blue DAPI staining outlines the insulitis mantle (L). Lower panels are enlargements of the fields outlined in the pictures to visualize the dense packing of the immune cells, mostly lymphocytes that formed the insulitis mantle. Bar, 100 μm in the pictures, 40 μm in the enlargements. **b)** All mice featured a similar percentage of islets with insulitis. **c)** However, a higher volume density of insulitis per unit pancreas volume was found after the glibenclamide treatments, due to the much larger number of islets surviving under these conditions. Data are mean + SEM values of the number of islets (b) or pancreas sections (c) indicated within the bars. * p< 0.05, ** p< 0.01, *** p< 0.001 vs control group, as tested by ANOVA.

In all NOD mice, the loss of beta cells associated with the development of a sizable insulitis (Figs 3A and 5A). Morphometric analysis showed that a similar proportion of islets was surrounded by a mantle of immune cells in the untreated and the two glibenclamide-treated groups (Fig 5B). However, the volume density of immune cells per pancreas was higher in the latter two groups than in the control group (Fig 5C), consistent with the much larger number of islets surviving in the pancreas of the NOD mice that received glibenclamide.

**Fig 6:**
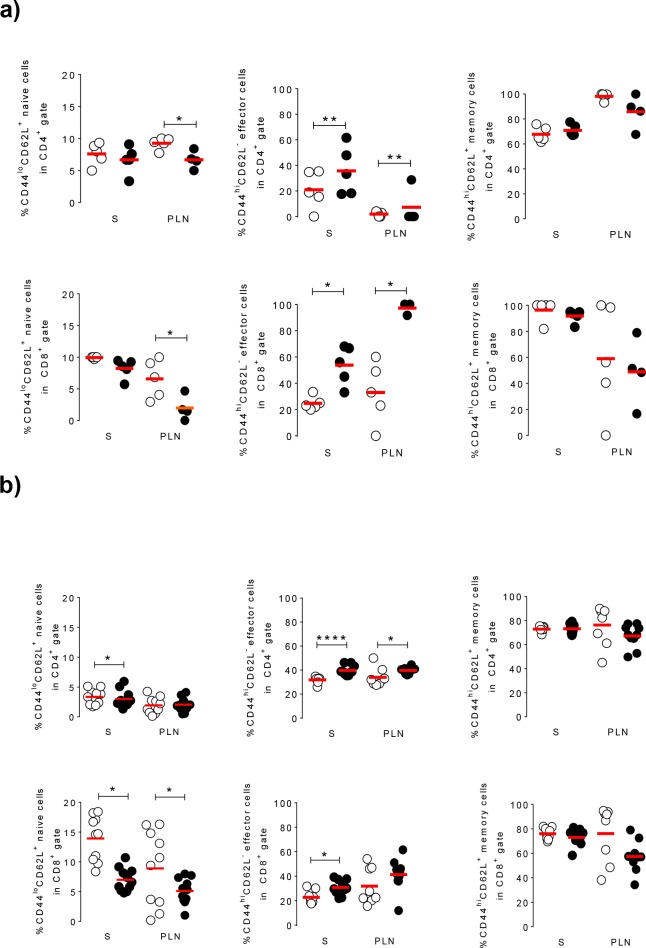
Glibenclamide alters the subsets of T cells in both spleen and pancreatic-draining lymph nodes. **a)** After 3 wks of treatment (mice 6 wk of age), FACS analysis showed that the treatment with glibenclamide dose 2 (solid symbols) increased the proportion of the effector subsets of CD44^hi^CD62L^-^ T cells, and decreased that of the naïve subset of CD44^lo^CD62L^+^ T cells in CD4^+^ (top panel) and CD8^+^ T cells (bottom panel), as compared to control values (open symbols). These changes took place in both spleen (S) and pancreatic-draining lymph nodes (PLN). **b)** Comparable observations were made after 6 wks of glibenclamide treatment (mice of 9 wks of age). Data are shown as scatters of individual values for the untreated control (open symbols, 5–10 mice) and the glibenclamide dose 2-treated group (solid symbols, 5–9 mice). Mean values are shown by the red lines. * p<0.05, ** p< 0.01, **** p<0.001 as compared to the untreated control group, using Mann-Whitney t-test.

After 3 wks of glibenclamide administration, the flow cytometry phenotyping by of cells from spleen and pancreatic draining lymph nodes revealed similar frequencies of total CD4^+^ and CD8^+^T cells in the mice of the control and the glibenclamide dose 2 groups (S2A Fig). Moreover, similar subsets of CD4^+^ (both CD25^+^ and CD25^-^) Tregs were observed in these two groups (S2B Fig). In contrast, further analysis of the CD4^+^ and CD8^+^ T cell populations revealed that glibenclamide at dose 2 increased the CD4^+^ and CD8^+^ effector T cells (CD^hi^CD62L^-^), and decreased the naïve CD4^+^ T cell subsets (CD44^lo^CD62L^+^) in both spleen and pancreas-draining lymph nodes (Fig 6A). Comparable observations were made after 6 wks of glibenclamide treatment (Fig 6B).

## Discussion

Glibenclamide (glyburide) is widely used in the treatment of several forms of diabetes [16,17], because it binds to the sulphonylurea receptors, which associate with the ATP-sensitive K^+^ channels of beta cells, thus stimulating insulin release [1]. The drug also activates other signalling mechanisms that are beneficial for beta cell function and survival, including those dependent on Cx36 [2–5]. We previously documented that the latter signalling improves the resistance of insulin-producing cells to a variety of cytotoxic conditions, including the *in vitro* exposure to Th1 cytokines [15,18] that prevail in the islet environment at the onset of type 1 diabetes [6]. Given that glibenclamide increases the expression and signaling of Cx36 [2–5], we exposed mouse insulin-producing MIN6 cells to the drug, in the presence of Th1 cytokines. We found that glibenclamide mitigated the cytokine-induced increase in cell apoptosis and, in parallel, reduced the cytokine-induced loss in the expression of Cx36, in keeping with the observations in primary islets [15] and other insulin-producing cell lines [18].

Previous studies have provided conflicting evidence about the pro- or anti-apoptotic role of sulphonylureas, and specifically of glibenclamide [9–11,13], which have been tentatively attributed to a variety of mechanisms. In view of this uncertainty, we revisited the diabetes-prone NOD mice, to assess whether the favourable *in vitro* effects of glibenclamide could help protecting beta cells *in vivo*, specifically against the initial autoimmune attack that triggers type 1 diabetes [6,19]. By initiating the drug administration in young animals, which had not yet developed insulitis and a type 1 diabetes phenotype [12,19], we found that glibenclamide provided a long-term (at least up to the term of this 25 wk-long experiment), *in vivo* protection against the loss of beta cells and the ensuing hyperglycaemia, which spontaneously developed within 16 weeks of age in the untreated NOD mice. This normoglycaemia was observed in spite of the development of a sizable insulitis, which had the same extent to that observed in the untreated NOD mice. The data extend the observations made with other sulphonylureas in both the NOD mouse and the BB rat autoimmune models which, however, reported a less complete protection, and did not investigate the mechanism underlying the effects on beta cells [7,8].

In this perspective, our novel *in vitro* observations on MIN6 cells, documenting a parallelism between the cytokine-induced apoptosis and loss of Cx36, suggest that this connexin is necessary for the glibenclamide effect, and provide a first plausible mechanistic scenario accounting for the beta cell protection by the drug. Indeed, glibenclamide promotes the *in vitro* and *in vivo* expression and function of Cx36 [2–5] which, in turn, benefits to both insulin secretion [4,5,20,21] and beta cell survival [15,18]. In the NOD model, this tentative conclusion is supported by the significantly lower levels of Cx36 in the whole pancreas of the untreated mice than in that of the glibenclamide-treated animals, which parallels major differences in the numerical and volume densities of the islets in the two animal groups, in spite of a comparable infiltration of the pancreas by immune cells. The data also document a preserved expression of Cx36 in the few residual islets of the untreated mice, and in the much more numerous islets of the glibenclamide-treated littermates, which remained normoglycaemic till the end of the experiment. This suggests that some Cx36 is required to sustain *in vivo* the beta cell survival. Future studies should validate this mechanism, e.g. by developing strains of NOD mice lacking or over-expressing Cx36, as we previously reported in non-diabetes prone mice [15].

Obviously, these considerations do not exclude that glibenclamide could also affect the immune mechanism which triggers beta cell apoptosis, as indicated by the changes in the phenotype of CD4^+^ and CD8^+^ T-cell subsets, that were observed in both the spleen and the lymph nodes draining the pancreas. Although the frequency of total CD4^+^ and CD8^+^ T cells of lymphoid organs was not altered by the glibenclamide treatment, the populations of effectors T cells (CD44^hi^CD62L^-^) was enriched by this treatment, while those of naive T cells (CD44^lo^CD62L^+^) were reduced. The data are consistent with a possible retention of the effector cells in the spleen and especially in the lymphatic nodes, as proposed in another setting [22]. These observations are in line with the reports that effector T cells which are not activated *in situ*, exit the inflamed organs via lymphatics and accumulate in the draining lymph nodes [23–25]. If the intracellular and molecular signalling cascades that coordinate effector T-cell retention versus egress remain largely unknown, the occurrence of insulitis in all the NOD mice we studied, indicates that the migration of autoimmune T cells into pancreatic islets was only partially impaired by glibenclamide. It is also possible that, in spite of unchanged subsets of Treg cells at the periphery, the glibenclamide treatment could restore and/or enhance the immunosuppressive function of these cells *in situ*, which may per se slow-down the beta cell attack leading to diabetes [26]. At this point, our data do not explain whether and how the immunological changes may contribute to the protective effect of glibenclamide [27,28]. Further studies, including *in vitro* suppression assays, mixed T cell transfer experiments, a complete characterization of T cell markers and an evaluation of cytokine production, should help validating this possibility. Additional studies should also establish whether the increased proportion of effector T cells we observed could be problematic, would a long-term glibenclamide treatment be considered in individuals susceptible to autoimmune diseases.

Our data provide evidence that exposure to glibenclamide, before MIN6 cells and NOD mice are exposed to molecules and cells that mediate an immune attack, is effective in preserving the mass and function of beta cells. Future studies should investigate whether the drug could also be beneficial when provided later, i.e. after the onset of the chain of events that increases *in vivo* beta cell apoptosis. Whether the present murine data warrant a clinical translation of the glibenclamide treatment also remains to be validated. Given that the highest glibenclamide dose we tested in NOD mice compares favorably with that recommended in patients [14], and that antibody screening can now predict within months the advance of type 1 diabetes in children [29,30], a plausible time window is available for testing the therapeutic value of the glibenclamide effects.

## Supporting Information

S1 Fig Flow cytometric gating strategy to distinguish T-cell subsets. A representative spleen analysis is shown, in which gated populations define viable CD3^+^CD4^+^ T cells (from left to right). **a)** CD4^+^ T cells are gated based on CD25 expression. CD25^+^ or CD25^-^ cells are further divided into Foxp3^+^ subsets. **b)** Viable CD3^+^CD4^+^ T cells are differentiated into naive (CD44^lo^CD62L^+^), effector (CD44^hi^CD62L^-^) and memory (CD44^hi^CD62L^+^) populations. A similar gating strategy was employed to screen for viable CD3^+^CD8^+^ T cells.
(TIF)

S2 Fig Glibenclamide dose 2 treatment does not alter the subsets of Treg cells subsets in lymphoid organs. a) After 3 wks of glibenclamide administration, phenotyping of cells from spleen (S) and pancreatic draining lymph nodes (PLN) revealed equal CD4^+^ and CD8^+^ T-cell frequencies. **b)** Similar subsets of CD4^+^CD25^+^ and CD25^-^Foxp3^+^ Tregs were also found in the control and the glibenclamide dose 2 groups. Mean values are shown by the red lines.
(TIF)
