# Supplementary figures and images for: Glibenclamide Prevents Diabetes in NOD Mice
Source: PLoS One. 2016 Dec 22;11(12):e0168839. doi: 10.1371/journal.pone.0168839 (PMC5178991; doi:10.1371/journal.pone.0168839)

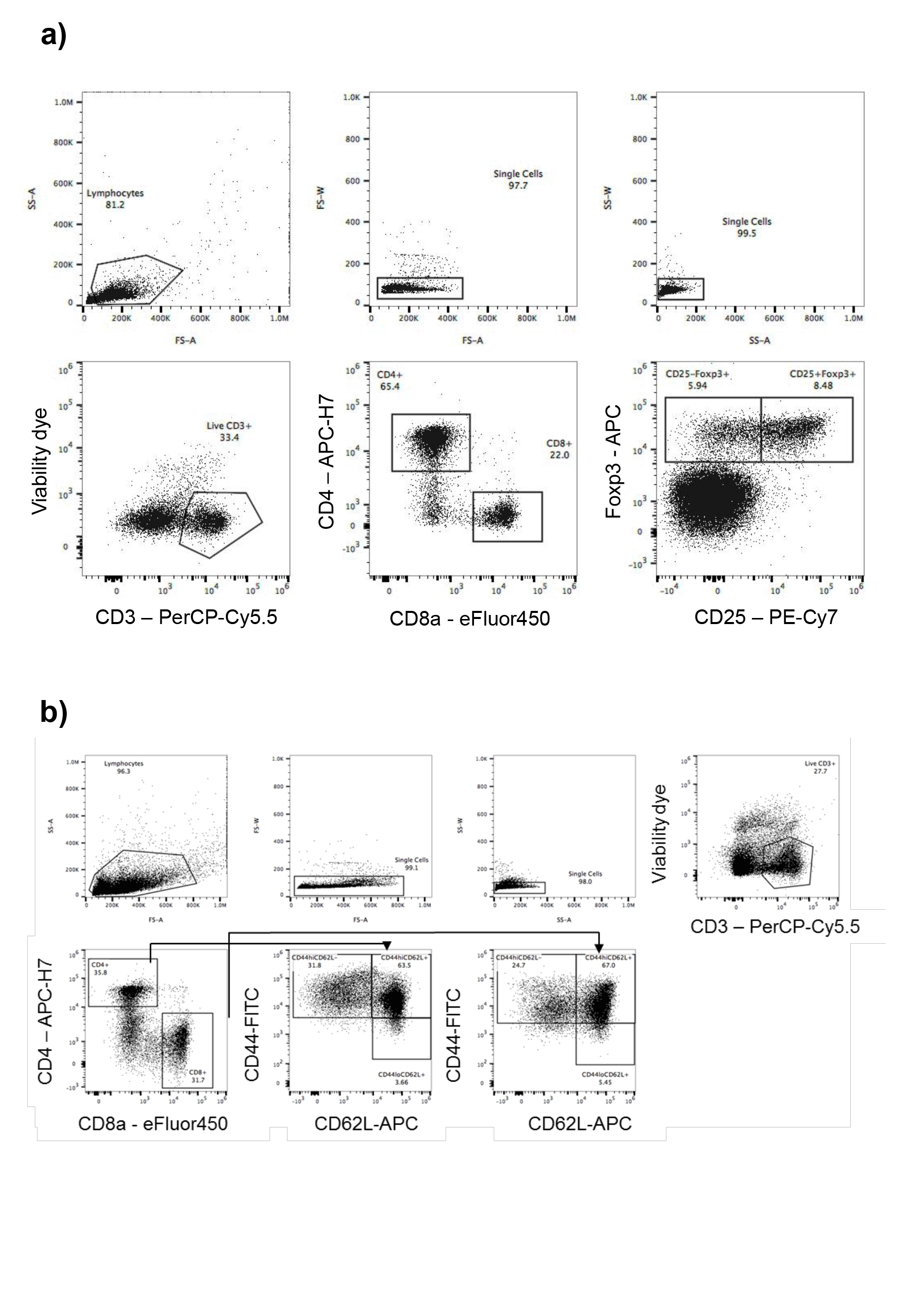

Supplement: S1 Fig — A representative spleen analysis is shown, in which gated populations define viable CD3+CD4+ T cells (from left to right). a) CD4+ T cells are gated based on CD25 expression. CD25+ or CD25- cells are further divided into Foxp3+ subsets. b) Viable CD3+CD4+ T cells are differentiated into naive (CD44loCD62L+), effector (CD44hiCD62L-) and memory (CD44hiCD62L+) populations. A similar gating strategy was employed to screen for viable CD3+CD8+ T cells. (TIF) [file pone.0168839.s001.tif]

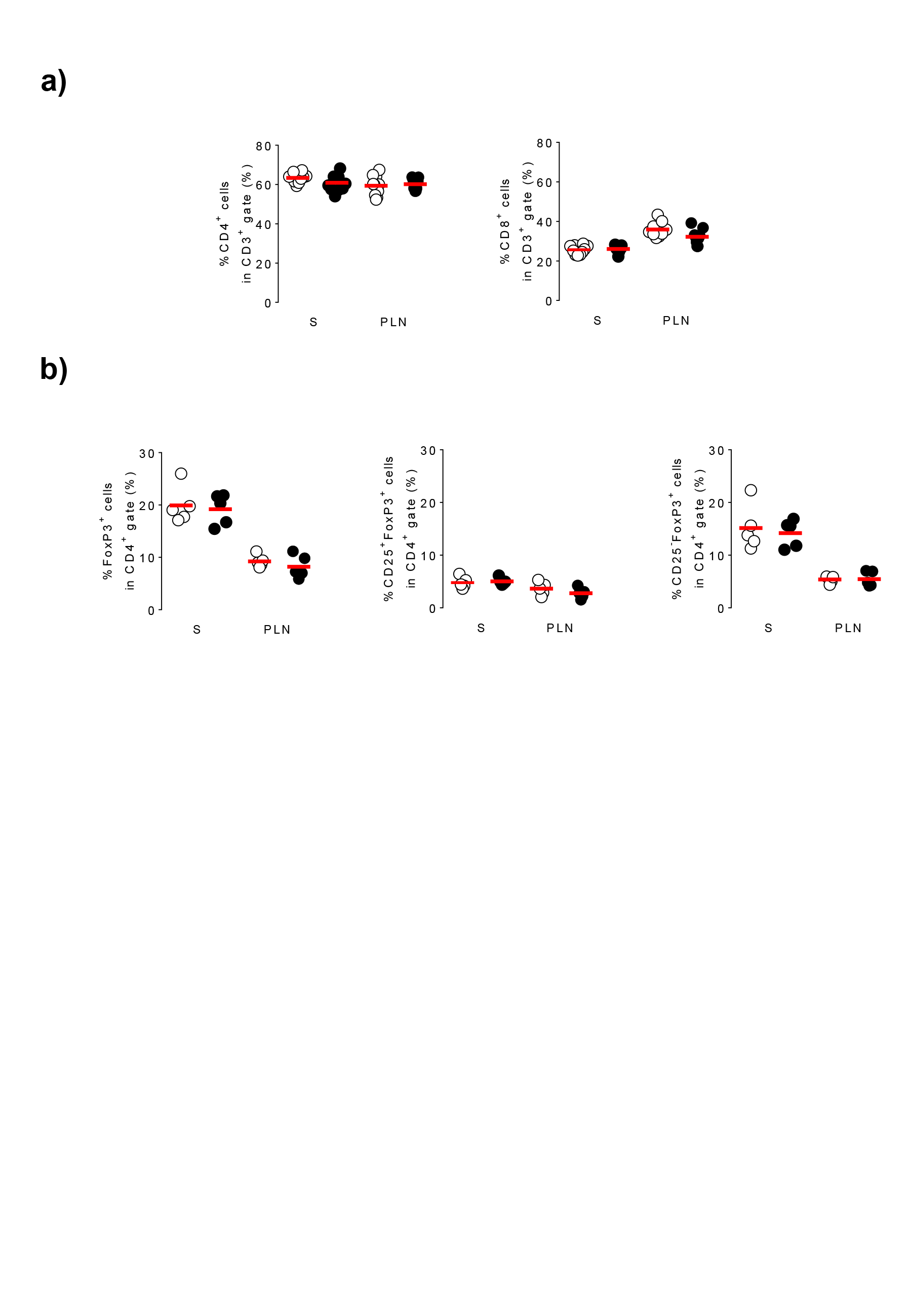

Supplement: S2 Fig — a) After 3 wks of glibenclamide administration, phenotyping of cells from spleen (S) and pancreatic draining lymph nodes (PLN) revealed equal CD4+ and CD8+ T-cell frequencies. b) Similar subsets of CD4+CD25+ and CD25-Foxp3+ Tregs were also found in the control and the glibenclamide dose 2 groups. Mean values are shown by the red lines. (TIF) [file pone.0168839.s002.tif]
